# Of Kindlins and Cancer

**DOI:** 10.15190/d.2016.6

**Published:** 2016-06-30

**Authors:** Edward F. Plow, Mitali Das, Katarzyna Bialkowska, Khalid Sossey-Alaoui

**Affiliations:** Joseph J. Jacobs Center for Thrombosis and Vascular Biology, Department of Molecular Cardiology, Lerner Research Institute, Cleveland Clinic, Cleveland, OH, USA

**Keywords:** Kindlins, integrins, cancer, FERMT genes, cancer therapy

## Abstract

Kindlins are 4.1-ezrin-ridixin-moesin (FERM) domain containing proteins. There are three kindlins in mammals, which share high sequence identity. Kindlin-1 is expressed primarily in epithelial cells, kindlin-2 is widely distributed and is particularly abundant in adherent cells, and kindlin-3 is expressed primarily in hematopoietic cells. These distributions are not exclusive; some cells express multiple kindlins, and transformed cells often exhibit aberrant expression, both in the isoforms and the levels of kindlins. Great interest in the kindlins has emerged from the recognition that they play major roles in controlling integrin function. In vitro studies, in vivo studies of mice deficient in kindlins, and studies of patients with genetic deficiencies of kindlins have clearly established that they regulate the capacity of integrins to mediate their functions. Kindlins are adaptor proteins; their function emanates from their interaction with binding partners, including the cytoplasmic tails of integrins and components of the actin cytoskeleton. The purpose of this review is to provide a brief overview of kindlin structure and function, a consideration of their binding partners, and then to focus on the relationship of each kindlin family member with cancer. In view of many correlations of kindlin expression levels and neoplasia and the known association of integrins with tumor progression and metastasis, we consider whether regulation of kindlins or their function would be attractive targets for treatment of cancer.

## 1. Introduction

Since the term “integrins” was coined more than 3 decades ago to designate a broadly distributed family of cell-surface adhesion receptors, the contributions of each of the 24 integrin family members in numerous physiological and pathological processes has remained a dominant theme in cell biology research. Indeed, it has now been broadly established that integrins have functions extending well beyond their primary roles in cell adhesion and migration; their contributions to bidirectional signaling, proliferation, gene regulation and cellular entry of pathogens have all been extensively documented. Research on integrins has extended from very basic investigations of their ligand binding repertoires and their three-dimensional structures to the clinical relevance of their antagonism as potential therapies. Indeed, clinical trials that led to approvals of several integrin directed drugs that have been used to treat patients with a variety of disorders, including thrombosis, cancer, ulcerative colitis and multiple sclerosis. Despite the breadth and depth of these studies, unanticipated findings regarding integrins and their functions have continued to emerge. Such a finding was the implication of kindlins into the integrin field starting about 10 years ago, but these cytosolic proteins are now accepted as pivotal regulators of integrin function^1-7^. A burgeoning subcontext of the relationship between kindlins and integrins is how their intercalation impacts cancer. Within the last 5 years, more than 70 publications have linked kindlins, integrins and cancer. In this review, we provide a brief synopsis of kindlin structure-function relationship, consider the latest findings on the role of kindlins in cancer, and then speculate as to the potential to target kindlins to inhibit cancer initiation, progression, metastasis and chemoresistance.

## 2. The kindlins

**Figure 1:**
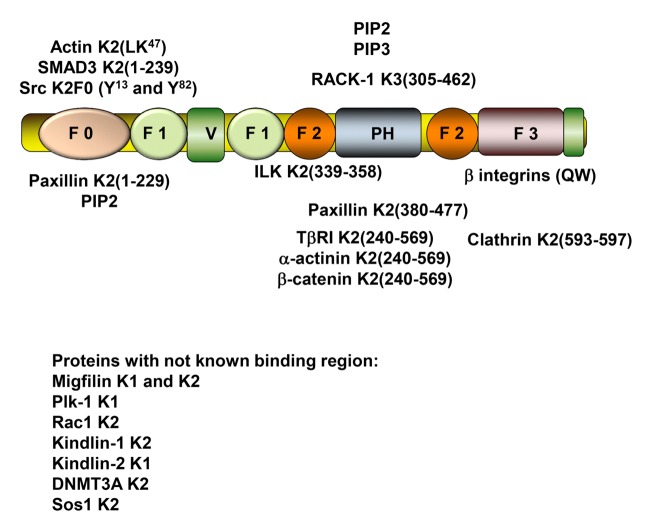
Binding partners of kindlins identified to date The numbers refer to the amino acid residues of the binding regions within the indicated kindlin. K1 (kindlin-1); K2 (kindlin-2); K3 (kindlin-3); ILK (integrin-linked kinase); RACK1 (receptor for activated C kinase 1); TβRI (TGF-β receptor I kinase); Plk-1 (polo-like kinase 1); DNMT3A-DNA (DNA (cytosine-5) methyltransferase 3A); Sos-1 (Son of sevenless homolog 1).

The prototypical structure of a kindlin is shown in**Figure 1**. Kindlins belong to the 4.1- ezrin-ridixin-moesin (FERM) domain containing protein family. Kindlins contain F1, F2 and F3 subdomains that typify FERM family members, and these subdomains are preceded by an N-terminal F0 subdomain in kindlins. A distinctive feature of kindlins is the insertion of a pleckstrin homology (PH) subdomain into the F2 subdomain (reviewed in ^4^). There are three kindlin family members in mammals, KINDLIN-1 (FERMT1; chromosome 20p12.3), KINDLIN-2 (FERMT2; chromosome 14q22.1) and KINDLIN-3 (FERMT3; chromosome 11q13.1). The three kindlins are highly homologous, sharing ~60% amino acid sequence identity^1^. Of the genomes analyzed, all metazoans, but no premetazoans, have at least one kindlin gene. Kindlin-2 is likely to have preserved the ancestral features of the kindlin family, and kindin-1 and kindlin-3 arose from duplications of the kindlin-2 gene. The ancestral kindlin itself appears to have evolved from duplication of the FERM domain in the N-terminal region of talin, and the two proteins share an overlapping function in integrin activation^8,9^. In humans, mutations leading to deficiencies of kindlin-1 cause Kindler Syndrome, which manifests with symptoms of skin fragility, blister formation, cutaneous atrophy, poikiloderma, and photosensitivity. Intestinal defects also occur frequently^10-12^. Mice deficient in kindlin-1 show similar phenotypes but the intestinal defects result in death shortly after birth^13^. Mutations in the kindlin-3 gene cause leukocyte adhesion deficiency type III (LAD III), which is characterized by high susceptibility to infections, spontaneous and episodic bleedings, and osteopetrosis^14-17^. These symptoms also occur in mice lacking kindlin-3 and result in mice that are only viable for a short time postnatally^18^. No deficiencies of kindlin-2 in humans have been reported to date, but disruption of kindlin-2 in mice results in embryonic lethality^19^. Mice with partial deficiency of kindlin-2, *Kindlin-2^+/-^*mice, exhibit no overt phenotype but display abnormal response in angiogenesis^20^, hemostasis^21^ and intracellular actin organization^22^. The three kindlins exhibit differences in their expression profiles: kindlin-1 is expressed mainly in epithelial cells; kindlin-2 is broadly expressed and is plentiful in endothelial cells, smooth muscle cells and fibroblasts^23^; and expression of kindlin-3 is restricted primarily to hematopoietic cells although it is also expressed in endothelial cells^24^. Several recent studies have, however, showed that aberrant expression of the kindlins occurs in several human cancers.

## 3. Kindlins as adaptor proteins

Kindlins are adaptor proteins. They lack intrinsic enzymatic activity but rather bind multiple effectors and thereby can build large multimolecular and multifunctional complexes. The binding sites for several kindlin binding partners have been positioned within the organization of the prototypic kindlin in**Figure 1**. Phospholipid binding sites exist in the F0^25,26^, F1^27,28^ and PH subdomains^29,30^. These interactions may target kindlins to membranes and optimize their orientation to execute other kindlin-dependent functions such as integrin activation. F0 also harbors binding sites for actin^22^; F2 contains the ILK binding site^31,32^; and, in addition to its phospholipid binding properties, the PH subdomain also contains a paxillin binding site^33^; and the F3 subdomain contains a clathrin^21^ and the primary integrin binding site (e.g.^34^). However, the primary function of kindlins, the capacity to support integrin activation, requires all subdomains of the kindlin^35^. The location of these binding sites has usually been established for one kindlin and may extrapolate to the other kindlin family members based on homology. Interactions of kindlins with ADAP^36^, RACK1^37^, scr^38^ and β-catenin^39^ also have been demonstrated. Some interactions may influence the function of an individual kindlin selectively as described in chapter 4. For example, ADAP can bind to both kindlin-2 and kindlin-3, but ADAP is restricted to hematopoietic cells^36^, where kindlin-3 exerts its major functions. Post-translational modifications of kindlins also occur, may be selective to specific kindlins and may influence the function of the modified kindlin^38,40^.

## 4. Functions of kindlins

*Integrin-dependent functions:* The most studied function of kindlins revolves around their role in integrin activation. Integrins can alter their affinity/avidity for their cognate ligands, a transition that is usually induced by stimulation of the integrin-bearing cell with agonists. Agonists may include G protein–coupled receptor ligands, growth factors, cytokines and shear stress (e.g. ^41-44^). Activation is particularly important for integrin-mediated responses of circulating blood cells, such as the adhesion of leukocytes to vascular cells^45,46^, of leukocytes to other blood cells^46-48^, or platelets to one another^49^. These responses do not occur in patients lacking kindlin-3; the integrin β1, β2 or β3 subclasses on hematopoietic cells do not undergo activation^16^. Integrins on adherent cells can also undergo activation although the changes are not as dramatic. Such integrin activation depends on inside-out signaling, which is a consequence of the binding of talin and kindlin to the cytoplasmic domain of integrins^7,50^. The detailed mechanisms of integrin activation have been the subject of reviews^6,51,52^ and are very dependent on the definition of “activation”. Is activation defined on a structural basis as straightening of the integrin legs from a bent to an extended conformation and/or opening of the headpiece, or is it the acquisition of functionally productive ligand binding^53^. Ligand binding and integrin clustering induce inside-out signaling. Frequently elicited consequences of outside-in signaling include cell spreading, changes in cell shape and gene expression. Kindlins are integrally involved in generating outside-in signals, which depends upon direct or indirect interactions with elements of actin cytoskeleton and the reorganization of focal adhesions, multimolecular signaling hubs within the cell.

*Integrin-independent functions of kindlins: *In a limited number of studies, functions have been assigned to kindlins that appear to be independent of their integrin binding activity. The integrin binding site of kindlins resides in their F3 (PTB-like) subdomain. Central to this binding function is a particular QW motif, Q^614^W^615^ in kindlin-2, and mutation of these residues to alanines markedly diminishes integrin binding activity. Using such mutant kindlins, the interaction of kindlin-2 with β-catenin to regulate Wnt signaling^39^ and kindlin-2 with clathrin to regulate cell surface expression of catabolic enzymes in endothelial cells^54^ have been identified as integrin-independent functions of kindlins. These mutations also demonstrated an integrin-independent role for kindlin-1 in Wnt signaling^55^. This strategy presumes that integrin binding to kindlins is completely disabled by the QW mutation. Clearly these mutations markedly reduce but may not completely disable integrin binding^34^.

## 5. Association of kindlins with cancer

The intimate interrelationship between integrins and cancer pathology has inevitably led to consideration of the role of kindlins in cancer. These efforts have identified associations of all three kindlin isoforms with cancers of many different tissues. In some cases, affected tissues is consistent with the distribution of the kindlin isoforms but expression levels are altered relative to the kindlin levels in the corresponding normal tissue (e.g., kindlin-1 and skin cancer^55^); whereas, in other cases, the particular kindlin is expressed at an unusual cell type (e.g. kindlin-3 sin breast cancer^56^).

### 5.1. Kindlin-1 and Cancer

One of the earliest evidences implicating kindlin-1 in cancer came from measurements of its mRNA expression levels. These levels were elevated in 60% of lung and 70% of colon cancers^57^. Kindlin-1 was also found to be associated with the pathology of glioma^58^. Kindlin-1 mRNA also was highly expressed in the pancreatic cancer cell lines and pancreatic cancer tissue^59^. Kindlin-1 protein was detected in the cytoplasm and membrane of the pancreatic cancer cells while normal ductal epithelial cells and stromal cells showed no expression. Sin and colleagues^60^ reported a role of kindlin-1 in the metastasis of tumors from various organs to the lungs and found that kindlin-1 expression correlated with a poor prognosis in both breast and lung adenocarcinoma^60^.

Despite these associations of kindlin-1 with cancer from many different organs, most studies documented aberrant kindlin-1 expression levels in cancers of epithelial origin^59-65^, consistent with its primary epithelial localization. In patients lacking kindlin-1 “Kindler Syndrome patients”, there is a suggestion of an increased risk of squamous cell carcinomas^66-71^; however, the rarity of the Kindler Syndrome precludes broad generalizations. Since kindlin-1 deficiency is lethal in mice due to intestinal manifestations. Rognoni and colleagues^55^ generated mice deficient in kindlin-1 in keratinocytes. These mice do exhibit an increased incidence of skin tumors that formed primarily as trichofolliculoma-like lesions and basal cell carcinomas, distinct from the tumors that were noted in patients with Kindler Syndrome^55^. Mechanistically, several interesting linkages have been uncovered between kindlin-1 and TGFβ activation, which exerts many opposing effects on the multiple steps associated with cancer progression and metastasis^72^. Gene expression microarray studies comparing the RNA profiles of TGFβ1-treated mammary epithelial cells with non-treated cells show that kindlin-1 is a TGFβ1 inducible gene^73^. Increase in kindlin-1 expression resulting from TGFβ1 treatment enhanced cell spreading and induced actin rearrangement, events correlated with the epithelial to mesenchymal transition (EMT), an important step in carcinogenesis^73^. TGF-β activation can be mediated by integrin αVβ6 and kindlin-1 can activate this integrin. Thus, an amplification loop may exist in which TGF-β enhances kindlin-1 synthesis and kindlin-1 enhances activation of TGF-β via αVβ6. High kindlin-1 levels have also been associated with high TGFβ-1 signaling in metastatic breast cancers^60^, and suppression of kindlin-1 in breast cancer cells significantly inhibited tumor growth and lung metastasis in an orthotopic mouse model. However, suppression of kindlin-1 in Kindler Syndrome patients may enhance cancer risk and, therefore, precludes broad generalizations.

### 5.2 Kindlin-2 and Cancer

Kindlin-2 expression has been found to be dysregulated in several cancer types: prostate^74-77^ breast^64,78-80^, lung^65,81^, colorectal cancer^82^, pancreas^83,84^ ovarian^85,86^, esophageal squamous cell carcinoma^87-89^, liver^90^, brain^91^, gastric cancer^92,93^, bladder^94^, and acute myeloid leukemia^95^. Given the association of kindlin-2 with several cancers of different origins, one can predict an important role that kindlin-2 may play in cancer pathogenesis. Kindlin-2 regulates tumor progression and metastasis by modulating several signaling pathways that are known to be critical for the regulation of cancer cell survival, proliferation, migration, invasion and metastasis. In fact, kindlin-2 has been associated with almost every hallmark of cancer^75^. In prostate cancer, kindlin-2 was found to promote the survival of prostate cancer cells by activating the nuclear factor kappa B (NFκB) survival pathway^74^. The invasive potential of prostate cancer cells was also activated as a result of the NFκB-mediated upregulation of matrix metalloproteinases expression and activity^74^. A positive feedback loop between kindlin-2 and TGF-β was identified to play a key role in promoting the progression and metastasis of pancreatic cancer^84^, which is characterized by its aggressiveness and the lack of effective therapies. While kindlin-2 expression levels were markedly elevated by Transforming Growth Factor 1 (TGF-β1) treatment, kindlin-2, in turn, activated the expression of TGF-β receptor I, a major component of TGF-β signaling^84^. Epithelial to mesenchymal transition, another significant hallmark of cancer, was found to be affected in esophageal squamous cell carcinoma as a result of dysregulation of kindlin-2^87^. Zhang and colleagues^87^ described how the loss of miR-200b, a well-established regulator of EMT, enhances invasion of esophageal squamous cell carcinoma cells by activating the Kindlin-2/integrin β1/AKT signaling pathway. Conversely, overexpression of miR-200b in these cells inhibited the integrin β1-AKT signaling by specific targeting of kindlin-2, which in turn suppressed invasion of ESCC cells^87^. Epidermal growth factor receptor (EGFR) is a known activator of cell proliferation, migration and tumor invasion in several cancers, including the one originating from the breast. A recent study by Guo et al.^80^ established a critical link between kindlin-2 and EGFR, where a physical interaction between the two was found to be necessary for the stabilization of EGFR and subsequent activation of the migration and invasion of breast cancer cells. Finally, several studies, including ours^75^, established a critical function of kindlin-2 to the modulation of chemoresistance, yet another major hallmark of cancer^72^. The Zhan lab^91^ described how kindlin-2 modulates the cisplatin-induced apoptosis and cell death of human glioma cells by regulating the AKT/JNK and AKT/p38 signaling pathways while the Zhang lab^77^ found kindlin-2 to activate the cisplatin-mediated cell death of prostate cancer cells through the regulation of the Bcl-xL cell death pathway. Our study has also established a major role for kindlin-2 in the regulation of the chemotherapy-induced cell death and apoptosis of metastatic castration-resistant prostate cancer, for which effective treatments have yet to be developed^75^. Loss of expression of kindlin-2 in prostate cancer cell lines significantly enhanced the sensitivity of these cells to docetaxel-induced apoptosis and cell death. Mechanistically, we found miR-138 to specifically target and inhibit kindlin-2 in prostate cancer cell lines, which resulted in dysregulation of the kindlin-2/β1-integrin pathway, thereby identifying a novel miR-138/kindlin-2/β1-integrin signaling axis that is critical for the modulation of sensitivity to chemotherapeutics. Therefore, targeted inhibition of kindlin-2 could be combined with chemotherapy to develop an effective treatment for prostate cancer^75^.

### 5.3 Kindlin-3 and cancer

Although Kindlin-3 is mainly expressed in the hematopoietic system, there are surprisingly only limited reports about its involvement in blood cell cancers. Kindlin-3 was found to be associated with the pathology of chronic myeloid leukemia^96^ and acute myeloid leukemia^95^. Qu and colleagues^96^ showed that kindlin-3 may regulate the proliferation of human chronic myeloid leukemia K562 cells through the regulation of c-Myc protein expression and controlled the tumor growth of these cells in a xenograft model^96^. In another study, Wu and colleagues reported that levels of kindlin-3 increased in patients with acute myeloid leukemia after complete remission^95^. Recent studies have identified kindlin-3 in solid tumors but its role as a tumor promoter or tumor suppressor is controversial. Our published study has identified kindlin-3 as a promoter of breast cancer progression and metastasis^56^. Kindlin-3 was over-expressed in both breast cancer cell lines and primary tumors, which is consistent with several Oncomine (www.oncomine.com) datasets, where kindlin-3 was in the top 3% of gene products elevated in breast cancer (p< 10^-12^)^56^. *In vitro* analyses determined that kindlin-3 stimulates breast cancer migration and invasion, and *in vivo* studies in mice showed that kindlin-3 stimulated tumor progression and metastasis. This function was traced to induction of tumor angiogenesis as a result of enhanced VEGF secretion and macrophage recruitment, downstream of Twist, which also activates the EMT program^56^. Nevertheless, a recent study by Djaafri and colleagues^97^ concluded that kindlin-3 has tumor suppressor function. The Djaafri study also included *in vitro* and *in vivo *supporting data and even included the MDA-MB-231 breast cancer cell line used by Sossey-Alaoui et al^56^. The basis for these differences is not clear although Djaafri et al. concluded kindlin-3 was a tumor promoter in K562 leukemic cells.

## 6. Kindlins as a cancer therapeutic target

Two independent studies have shown that reduction in kindlin-2 levels, either with siRNA that targets kindlin mRNA directly or by overexpression of miRNAs that reduce kindlin expression, sensitizes tumor cell lines to chemotherapeutic agents such as docetaxel^75^ and cisplatin^77,91^. Knockdown of kindlin-2 would also blunt certain properties of tumor cells that are associated with cancer progression, such as angiogenesis, invasion, recruitment of tumor promoting macrophages and formation of invadopodia. Rather than at the gene expression level, it may be feasible to selectively inhibit selective functions of a specific kindlin family member. For example, most of the functions of kindlin in tumor cell biology revolve around their interaction with the cytoplasmic tails of integrins. Specific short peptide sequences within integrin β tails have been located that are necessary for kindlins to exert their integrin regulatory activity^35^. Peptides or peptidomimetics of these sequences that block kindlin binding to integrins could be delivered into cells and blunt responses. These sequences could be tailored to block interaction of kindlins with specific integrins, or individual kindlins with specific integrins. However, knocking down kindlins may not be the panacea for cancer treatment. In a recent publication, Rognoni et al.^55^ provided a comprehensive and valuable list of the reports in which kindlin levels were altered in tumors. In most of the listed studies, the levels of the kindlin under investigation were increased, but in six of the 20 reports, reduced levels of kindlin were associated with cancer or a transformed cell phenotype. As noted above, the absence of kindlin-1 in Kindler Syndrome patients appears to be associated with an increase in cancer. Thus, just as there is a TGF-β paradox, there may be a “kindlin paradox” and the value of a kindlin targeted therapy may need to be context specific. Despite this uncertainty, the relationship between kindlins and cancer remains an important interrelationship to dissect, and manipulation of kindlin functions and/or levels may provide insights into tumorogenesis and may ultimately offer a therapeutic strategy.

## KEY POINTS

**◊** **Kindlins are FERM domain adaptor proteins**

**◊ Kindlins are critical regulators of integrin function**

**◊** **Expression of each of the three kindlins is altered in many different human cancers**

**◊** **Kindlins can influence the growth and metastatic properties of cancer cells in in vivo models and modulate sensitivities to chemotherapies**
